# The Added Value of Water Footprint Assessment for National Water Policy: A Case Study for Morocco

**DOI:** 10.1371/journal.pone.0099705

**Published:** 2014-06-11

**Authors:** Joep F. Schyns, Arjen Y. Hoekstra

**Affiliations:** Twente Water Centre, University of Twente, Enschede, The Netherlands; Centro de Investigacion Cientifica y Educacion Superior de Ensenada, Mexico

## Abstract

A Water Footprint Assessment is carried out for Morocco, mapping the water footprint of different activities at river basin and monthly scale, distinguishing between surface- and groundwater. The paper aims to demonstrate the added value of detailed analysis of the human water footprint within a country and thorough assessment of the virtual water flows leaving and entering a country for formulating national water policy. Green, blue and grey water footprint estimates and virtual water flows are mainly derived from a previous grid-based (5×5 arc minute) global study for the period 1996–2005. These estimates are placed in the context of monthly natural runoff and waste assimilation capacity per river basin derived from Moroccan data sources. The study finds that: (i) evaporation from storage reservoirs is the second largest form of blue water consumption in Morocco, after irrigated crop production; (ii) Morocco’s water and land resources are mainly used to produce relatively low-value (in US$/m^3^ and US$/ha) crops such as cereals, olives and almonds; (iii) most of the virtual water export from Morocco relates to the export of products with a relatively low economic water productivity (in US$/m^3^); (iv) blue water scarcity on a monthly scale is severe in all river basins and pressure on groundwater resources by abstractions and nitrate pollution is considerable in most basins; (v) the estimated potential water savings by partial relocation of crops to basins where they consume less water and by reducing water footprints of crops down to benchmark levels are significant compared to demand reducing and supply increasing measures considered in Morocco’s national water strategy.

## Introduction

Morocco is a semi-arid country in the Mediterranean facing water scarcity and deteriorating water quality. The limited water resources constrain the activities in different sectors of the economy of the country. Agriculture is the largest water consumer and withdrawals for irrigation peak in the dry period of the year, which contributes to low surface runoff and desiccation of streams. Currently, 130 reservoirs are in operation to deal with this mismatch in water demand and natural water supply and to serve for generation of hydroelectricity and flood control [1]. Groundwater resources also play an important role in the socio-economic development of the country, in particular by ensuring the water supply for rural communities [2]. However, a large part of the aquifers is being overexploited and suffer from deteriorating water quality by intrusion of salt water, caused by the overexploitation, and nitrates and pesticides that leach from croplands, caused by excessive use of fertilizers. Surface water downstream of some urban centres is also polluted, due to untreated wastewater discharges.

In 1995, the Moroccan Water Law (no. 10–95) came into force and introduced decentralized integrated water management and rationalisation of water use, including the user-pays and polluter-pays principles. It also dictates the development of national and river basin master plans [3], which are elaborated in accordance with the national water strategy. To cope with water scarcity and pollution, the national water strategy includes action plans to reduce demand, increase supply and preserve and protect water resources [1]. It also proposes legal and institutional reforms for proper implementation and enforcement of these actions. Demand management focuses on improving the efficiency of irrigation and urban supply networks and pricing of water to rationalise its use. Plans to increase supply include the construction of more dams and a large North-South inter-basin water transfer, protection of existing hydraulic infrastructure, desalinization of sea water and reuse of treated wastewater.

Although the national water strategy considers options to reduce water demand in addition to options to increase supply, it does not include the global dimension of water by considering international virtual water trade, nor does it consider whether water resources are efficiently allocated based on physical and economic water productivities of crops (the main water consumers). Analysis of the water footprint of activities in Morocco and the virtual water trade balance of the country therefore might reveal new insights to alleviate water scarcity.

The concept of water footprint was introduced by Hoekstra [4]; this subsequently led to the development of Water Footprint Assessment as a distinct field of research and application [5], [6]. The water footprint is an indicator of freshwater use that looks not only at direct water use of a consumer or producer, but also at the indirect water use. As such, it provides a link between human consumption and human appropriation of freshwater systems. Water Footprint Assessment refers to a variety of methods to quantify and map the water footprint of specific processes, products, producers or consumers, to assess the environmental, social and economic sustainability of water footprints at catchment or river basin level and to formulate and assess the effectiveness of strategies to reduce water footprints in prioritized locations. The water footprint of a product is the volume of freshwater used to produce the product, measured over the full supply chain [6]. Three different components of a water footprint are distinguished: green, blue and grey. The green water footprint is the volume of rainwater evaporated or incorporated into the product. Blue water refers to the volume of surface- or groundwater evaporated, incorporated into the product or returned to another catchment or the sea. The grey water footprint relates to pollution and is defined as the volume of freshwater that is required to assimilate the load of pollutants given natural background concentrations and existing ambient water quality standards [6]. The total freshwater volume consumed or polluted within the territory of a nation as a result of activities within the different sectors of the economy is called the water footprint of national production. International trade of products creates ‘virtual water flows’ leaving and entering a country. The virtual-water export from a nation refers to the water footprint of the products exported. The virtual-water import into a nation refers to the water footprint of the imported products.

Several authors have assessed the water footprint and virtual water trade balance of nations and regions and state the relevance of the tool for well-informed water policy on the national and river basin level [7]–[10]. In a case study for a Spanish region, Aldaya *et al.* [10] conclude that water footprint analyses can provide a transparent framework to identify potentially optimal alternatives for efficient water use at the catchment level and that this can be very useful to achieve an efficient allocation of water and economic resources in the region. Chahed *et al.* [8] state that integration of all water resources at the national scale, including the green water used in rain-fed agriculture and as part of the foodstuffs trade balance, is essential in facing the great challenges of food security in arid countries.

The objective of this study is to explore the added value of analysing the water footprint of activities in Morocco and the virtual water flows from and to Morocco in formulating national water policy. The study includes an assessment of the water footprint of activities in Morocco (at the river basin level on a monthly scale) and the virtual water trade balance of the country and, based on this, response options are formulated to reduce the water footprint within Morocco, alleviate water scarcity and allocate water resources more efficiently. Results and conclusions from the Water Footprint Assessment are compared with the scope of analysis of, and action plans included in Morocco’s national water strategy and river basin plans in order to address the added value of Water Footprint Assessment relative to these existing plans.

The water footprint of Morocco has not been assessed previously on the river basin level on a monthly scale. Morocco has been included in a number of global studies, but these studies did not analyse the spatial and temporal variability of the water footprint within the country [11]–[13]. Furthermore, this study is the first to include specific estimates of the evaporative losses from the irrigation supply network and from storage reservoirs as part of a comprehensive Water Footprint Assessment. Finally, it is new in providing quantitative estimates of the potential water savings by partial relocation of crop production to regions with lower water consumption per ton of crop by means of an optimization and by reducing water footprints of crops down to benchmark levels.

Several insights and response options emerged from the Water Footprint Assessment, which are currently not considered in the national water strategy of Morocco and the country’s river basin plans. Therefore, Water Footprint Assessment is considered to have an added value for formulating national water policy in Morocco.

## Method and Data

### Water Footprint of Morocco’s Production

**Figure 1:**
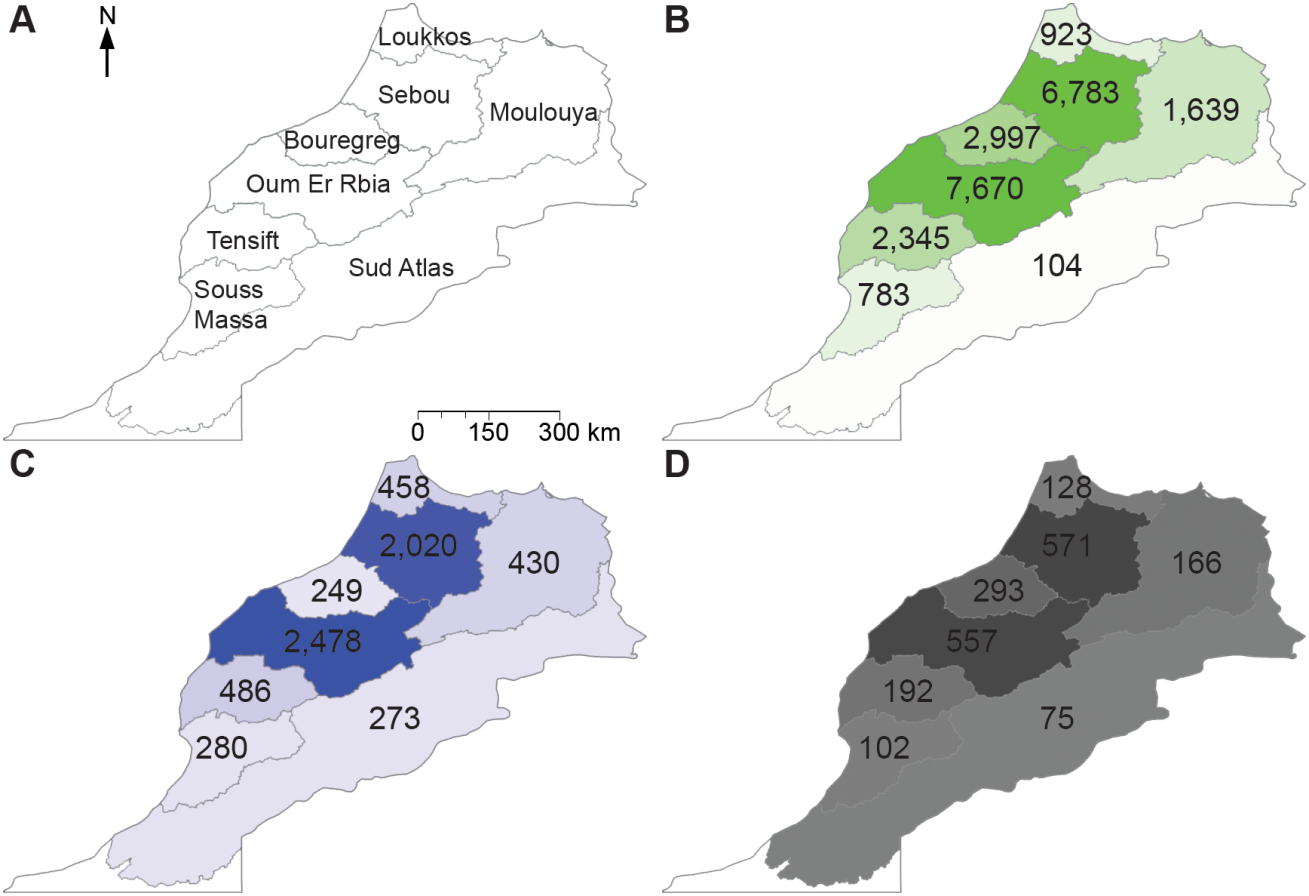
Water footprint of Morocco’s production per river basin. Period: 1996–2005. Morocco’s river basins (A) and total green (B), blue (C) and grey (D) water footprint of Morocco’s production per river basin (in Mm^3^/yr).

This study follows the terminology and methodology developed by Hoekstra *et al.* [6]. The water footprint of Morocco’s production is estimated at river basin level on a monthly scale for the activities included in Table 1. The river basins are chosen such that they coincide with the action zones of Morocco’s river basin agencies (Figure 1A). Due to data limitations, the grey water footprint is analysed on an annual scale and the water footprints of grazing and animal water supply are analysed at national and annual level. The study considers the average climate, production and trade conditions over the period 1996–2005. The water footprints of agriculture, industry and households are obtained from Mekonnen and Hoekstra [13], [14], who estimated these parameters globally at a 5 by 5 arc minute spatial resolution. The annual blue water footprint estimates for industries and households by Mekonnen and Hoekstra [13] are distributed throughout the year according to the monthly distribution of public water supply obtained from Ministry EMWE (unpublished data 2013). These distributions are available for the basins Loukkos, Sebou, Bouregreg and Oum Er Rbia. For the other basins an average of these distributions is taken.

**Table 1:** Water footprint estimates included in this study.

Water footprint of	Components	Period	Source
Crop production	Green, blue, grey	1996–2005	[14]
Grazing	Green	1996–2005	[13]
Animal water supply	Blue	1996–2005	[13]
Industrial production	Blue, grey	1996–2005	[13]
Domestic water supply	Blue, grey	1996–2005	[13]
Storage reservoirs	Blue	-	Own elaboration
Irrigation water supply network	Blue	1996–2005	Own elaboration

The monthly water footprint of storage reservoirs (in m^3^/yr) is calculated as the open water evaporation (in m/yr) times the surface area of storage reservoirs (in m^2^). Data on open water evaporation from the reservoirs in the basins Loukkos, Sebou, Bouregreg and Oum Er Rbia is obtained from Ministry EMWE (unpublished data 2013) and for the other basins from a model simulation with the global hydrological model PCR-GLOBWB carried out by Sperna Weiland *et al*. [15]. The surface area of reservoirs at upper storage level is derived from Ministry EMWE (unpublished data 2013) and FAO [16]. Since storage levels vary throughout the year (and over the years), and reservoir areas accordingly, this gives an overestimation of the evaporation from reservoirs. To counteract this overestimation, but due to lack of data on monthly storage level and reservoir area, for all months a fraction of the evaporation at upper storage level (43%) is taken as estimate of the water footprint of storage reservoirs. This fraction represents the average reservoir area as fraction of its area at upper storage level, calculated as the average over the reservoirs in the basins Loukkos, Sebou, Bouregreg and Oum Er Rbia for which data on surface area at different reservoir levels is available from Ministry EMWE (unpublished data 2013).

The water footprint of the irrigation supply network refers to the evaporative loss in the network and is estimated based on a factor *K*, which is defined as the ratio of the blue water footprint of the irrigation supply network to the blue surface water footprint of crop production at field level (i.e. crop evapotranspiration of irrigation water stemming from surface water). The blue water footprint of crop production at field level is taken from Mekonnen and Hoekstra [14] and the split to surface water is made according to the fraction of irrigation water withdrawn from surface water (as opposed to groundwater) per river basin based on data from the associated river basin plans. *K* is calculated as:
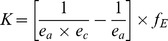
in which *e_a_* represents the field application efficiency, *e_c_* the irrigation canal efficiency and *f_E_* the fraction of losses in the irrigation canal network that evaporates (as opposed to percolates). The irrigation efficiencies *e_a_* and *e_c_* are estimated based on data from a local river basin agency and FAO [17]. The value of *f_E_* is assumed at fifty percent. The resultant *K* for Morocco’s irrigated agriculture as a whole is 15%, i.e. the evaporative loss from the irrigation water supply network represents a volume equal to 15% of the blue surface water footprint of crop production at field level on average.

### Water Footprint and Economic Water and Land Productivity of Crops

The water footprint of crops per unit of production (in m^3^/ton) is calculated by dividing the water footprint per hectare (in m^3^/ha/yr) by the yield (in ton/ha/yr), for which data are obtained from Mekonnen and Hoekstra [14]. Economic water productivity (in US$/m^3^) represents the economic value of farm output per unit of water consumed and is calculated as the average producer price for the period 1996–2005 (in US$/ton) obtained from FAO [18] divided by the green plus blue water footprint (in m^3^/ton). Similarly, economic land productivity (in US$/ha) represents the economic value of farm output per hectare of harvested land and is calculated as the same producer price multiplied by crop yield (in ton/ha), which is also obtained from Mekonnen and Hoekstra [14].

### Virtual Water Flows and Associated Economic Value

Green, blue and grey virtual water flows related to Morocco’s import and export of agricultural and industrial commodities for the period 1996–2005 are obtained from Mekonnen and Hoekstra [13], who estimated these flows at a global scale based on trade matrices and water footprints of traded products at the locations of origin. The virtual water export that originates from domestic water resources (another part is re-export) is estimated based on the relative share of the virtual water import to the total water budget:
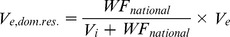
in which *WF_national_* is the water footprint within the nation, *V_i_* the virtual water import and *V_e_* the virtual water export.

The average earning per unit of water exported (in US$/m^3^) is calculated by dividing the value of export (in US$/yr) by virtual water export (in m^3^/yr). Similarly, the cost per unit of virtual water import is calculated by dividing the import value (in US$/yr) by virtual water import (in m^3^/yr). The average economic value of import and export for the period 1996–2005 are derived from the Statistics for International Trade Analysis (SITA) database from the International Trade Centre [19].

### Water Footprint versus Water Availability and Waste Assimilation Capacity

To assess the environmental sustainability of the water footprint within Morocco, the total blue (surface- plus groundwater) water footprint of production is placed in the context of monthly natural runoff and the groundwater footprint in the context of annual groundwater availability. The water needed to assimilate the nitrogen fertilizers that reach the water systems due to leaching is compared with the waste assimilation capacity of aquifers.

The groundwater footprint is calculated by splitting the blue water footprint of crop production, industrial production and domestic water supply according to the fraction withdrawn from groundwater per river basin based on data from the associated river basin plans. Assuming that none of the water abstracted from groundwater for industrial production and domestic water supply returns (clean) to the groundwater in the same period of time, the groundwater footprints of these purposes are increased to equal water withdrawal (as opposed to consumption) by dividing them by the consumptive fractions assumed by Mekonnen and Hoekstra [13]: 5% for industries and 10% for households.

Long-term average monthly natural runoff (1980–2011) for the river basins of Loukkos, Sebou, Bouregreg and Oum Er Rbia is derived from Ministry EMWE (unpublished data 2013). Natural runoff is estimated as the inflow of reservoirs. It is considered undepleted runoff, since large-scale blue water withdrawals come from the reservoirs. For the other basins, long-term average annual natural runoff is derived from the river basin plans for the respective river basins and subsequently distributed over the months according to intra-annual rainfall patterns [20], [21] or monthly natural discharge [22]. Due to lack of data, for the Souss Massa basin the same monthly variation is applied as for the adjacent Tensift basin. Groundwater availability is assessed on river basin scale and defined as the recharge by percolation of rainwater and from rivers, minus the direct evaporation from aquifers. These data are obtained from the river basin plans and from Laouina [23] for the basin of Souss Massa.

Blue water scarcity is defined as the ratio of the total blue water footprint in a catchment over the blue water availability in that catchment [6]. In this study, this ratio is calculated as the total blue water footprint to monthly natural runoff and as the groundwater footprint to annual groundwater availability. Following Hoekstra *et al*. [24], blue water scarcity values have been classified into four levels of water scarcity. The classification in this study corresponds with their classification, with the note that the current study does not account for environmental flow requirements in the definition of blue water availability, since they are generally not considered in Morocco’s river basin plans and local studies on the level of these requirements are lacking. This is compensated for by using stricter threshold values for the different scarcity levels, so that the resultant scheme is equivalent to that of Hoekstra *et al*. [24]:

- low blue water scarcity (<0.20): the blue water footprint is lower than 20% of natural runoff; river runoff is unmodified or slightly modified.
- moderate blue water scarcity (0.20–0.30): the blue water footprint is between 20 and 30% of natural runoff; runoff is moderately modified.
- significant blue water scarcity (0.30–0.40): the blue water footprint is between 30 and 40% of natural runoff; runoff is significantly modified.
- severe water scarcity (>0.40): the monthly blue water footprint exceeds 40% of natural runoff, so runoff is seriously modified.

The water pollution level is defined as the total grey water footprint in a catchment divided by the waste assimilation capacity [6]. In other words, it shows the fraction of actual runoff that is required to dilute pollutants in order to meet ambient water quality standards. A water pollution level greater than 1 means that ambient water quality standards are violated. The nitrate-related grey water footprint of crop production as computed in this study is assumed to mostly contribute to groundwater pollution and is therefore compared with the waste assimilation capacity of groundwater. As a measure of the latter, we use the actual groundwater availability, calculated as (natural) groundwater availability minus the groundwater footprint.

### Relocation of Crop Production and Reducing Water Footprints of Crops to Benchmark Levels

The potential water savings by changing the pattern of crop production across river basins (which is possible due to spatial differences in crop water use) are quantified by means of an optimization model. The total green plus blue water footprint of twelve main crops in the country (in Mm^3^/yr) is minimized by changing the spatial pattern of production (in ton/yr) over the river basins under constraints for production demand (in ton/yr) and land availability (in ha/yr). The analysed crops are: almonds, barley, dates, grapes, maize, olives, oranges, sugar beets, sugar cane, mandarins, tomatoes and wheat. Results are compared with a base case, which corresponds with the average green plus blue water footprint of the analysed crops over the period 1996–2005. Land availability is restricted per river basin and taken equal to the average harvested area in the period 1996–2005 obtained from Mekonnen and Hoekstra [14]. Two cases are distinguished: 1) all crops can be relocated; 2) only annual crops (barley, maize, sugar beets, tomatoes and wheat) can be relocated, perennials cannot. For both cases, the restriction is imposed that the total national production per crop (in ton/yr) should be equal to (or greater than) the total national production of the crop in the base case, which is defined as the average production in the period 1996–2005 obtained from Mekonnen and Hoekstra [14].

Additionally, an assessment is made of the potential water savings by reducing the water footprints of the twelve main crops down to certain benchmark levels. For each basin and crop a benchmark is set in the form of the lowest water consumption (green plus blue) of that crop which is achieved in a comparable river basin in Morocco. In this case, basins are considered comparable when the reference evapotranspiration (ET_0_ in mm/yr) is in the same order of magnitude (see Table 2). Reference evapotranspiration expresses the evaporating power of the atmosphere at a specific location (and time of the year) and does not consider crop characteristics and soil factors [6]. Differences in soil and development conditions are thus not accounted for.

**Table 2:** Comparison of river basins based on reference evapotranspiration (ET_0_ in mm/yr, period: 1961–1990).

No.	River basin	ET_0_ (mm/yr)	Considered comparable with no.
1	Sud Atlas	1,652	-
2	Souss Massa	1,450	3
3	Moulouya	1,409	2
4	Tensift	1,389	5
5	Oum Er Rbia	1,387	4
6	Sebou	1,266	7,8
7	Bouregreg	1,239	6,8
8	Loukkos	1,212	6,7

Source: ET_0_ from FAO [31].

## Results

### Water Footprint of Morocco’s Production

The total water footprint of Morocco’s production in the period 1996–2005 was 38.8 Gm^3^/yr (77% green, 18% blue, 5% grey), see Table 3. Crop production is the largest contributor to this water footprint, accounting for 78% of all green water consumed, 83% of all blue water consumed (evaporative losses in irrigation water supply network included) and 66% of the total volume of polluted water. Evaporative losses from storage reservoirs are estimated at 884 Mm^3^/yr, which is 13% of the total blue water footprint within Morocco. For most reservoirs, these losses are ultimately linked to irrigated agriculture and in some cases potable water supply.

**Table 3:** Water footprint of Morocco’s production in the period 1996–2005 (in Mm^3^/yr).

Water footprint of	Green	Blue	Grey	Total
Crop production^a)^	23,245	5,097	1,378	29,719
Grazing^a)^	6,663	-	-	6,663
Animal water supply^a)^	-	151	-	151
Industrial production^a)^	-	18	69	88
Domestic water supply^b)^	-	125	640	765
Storage reservoirs^b)^	-	884	-	884
Irrigation water supply network^b)^	-	549	-	549
Total water footprint	29,908	6,824	2,087	38,819

Source: ^a)^ [13], ^b)^ Own elaboration.

Largest water footprints (green, blue and grey) are found in the basins Oum Er Rbia and Sebou, the basins containing the main agricultural areas of Morocco (see Figure 1B–D). Together, these two basins account for 63% of the total water footprint of national production. In general, the green water footprint is largest in the rainy period December–May, while the blue water footprint is largest in the period April–September when irrigation water use increases.

In the basins Bouregreg and Loukkos, evaporation from storage reservoirs accounts for 45% and 55% of the total blue water footprint, respectively. Irrigated agriculture is the largest blue water consumer in the other basins, but evaporation from storage reservoirs is also significant in these basins. Main irrigated crops in the Oum Er Rbia basin are maize, wheat, olives and sugar beets, which together account for 60% of the total irrigation water consumed in the period 1996–2005. In the basin of Sebou, 56% of the blue water footprint of crop production relates to the irrigation of wheat, olives, sugar beets, sugar cane and sunflower seed.

### Water Footprint and Economic Water and Land Productivity of Main Crops

**Figure 2:**
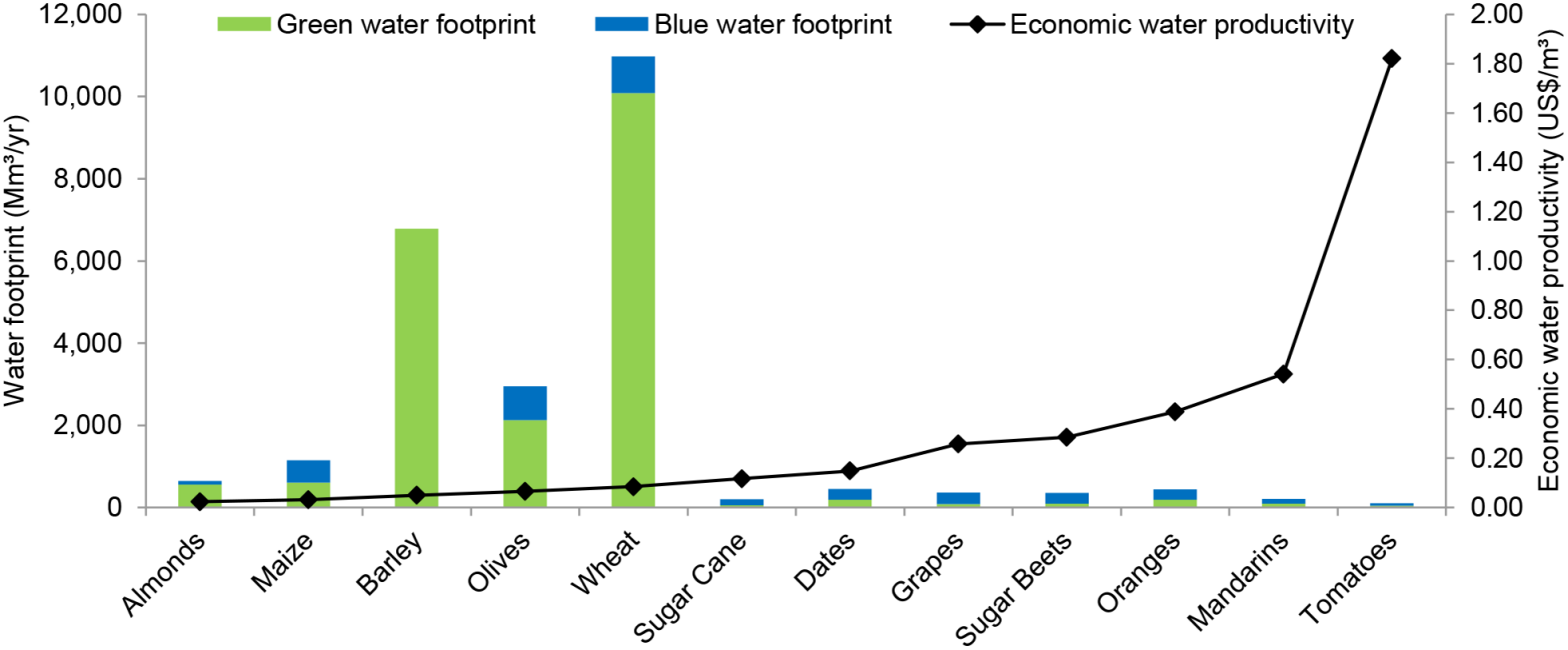
Economic water productivity and green and blue water footprint of main crops in Morocco. Period: 1996–2005. Source: Water footprint from Mekonnen and Hoekstra [14], producer prices from FAO [18].

In the period 1996–2005, most green water was consumed by the production of wheat, barley and olives (Figure 2). The largest blue water footprints relate to the production of wheat, olives and maize. For wheat, the number one blue water consuming crop, the blue water footprint was largest in the period March–May and peaked in April.

**Figure 3:**
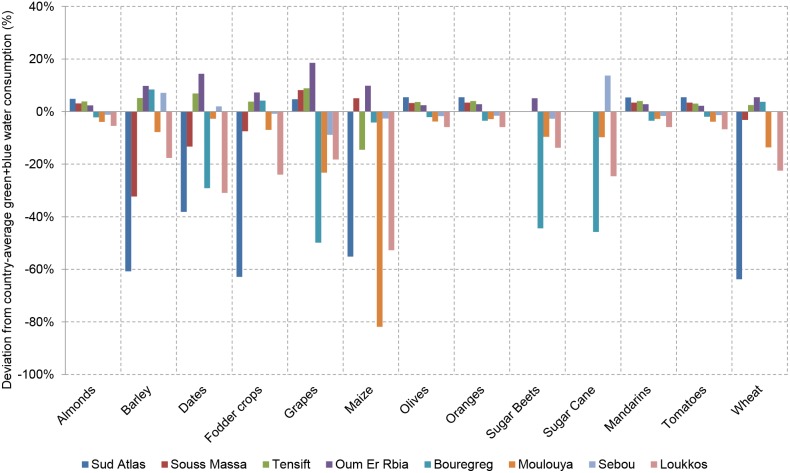
Variation in green plus blue water consumption (in m^3^/ton) across river basins. Period: 1996–2005.

Water consumption of crops (green plus blue, in m^3^/ton) varies significantly per river basin due to differences in climatic conditions. In general, water consumption of crops is above country-average in the basins Oum Er Rbia and Tensift and below country-average in the northern basins Bouregreg, Sebou, Loukkos and Moulouya (Figure 3). In the basins Sud Atlas and Souss Massa the picture is not so clear, with some crops having above and others below country-average water footprints (in m^3^/ton).

**Figure 4:**
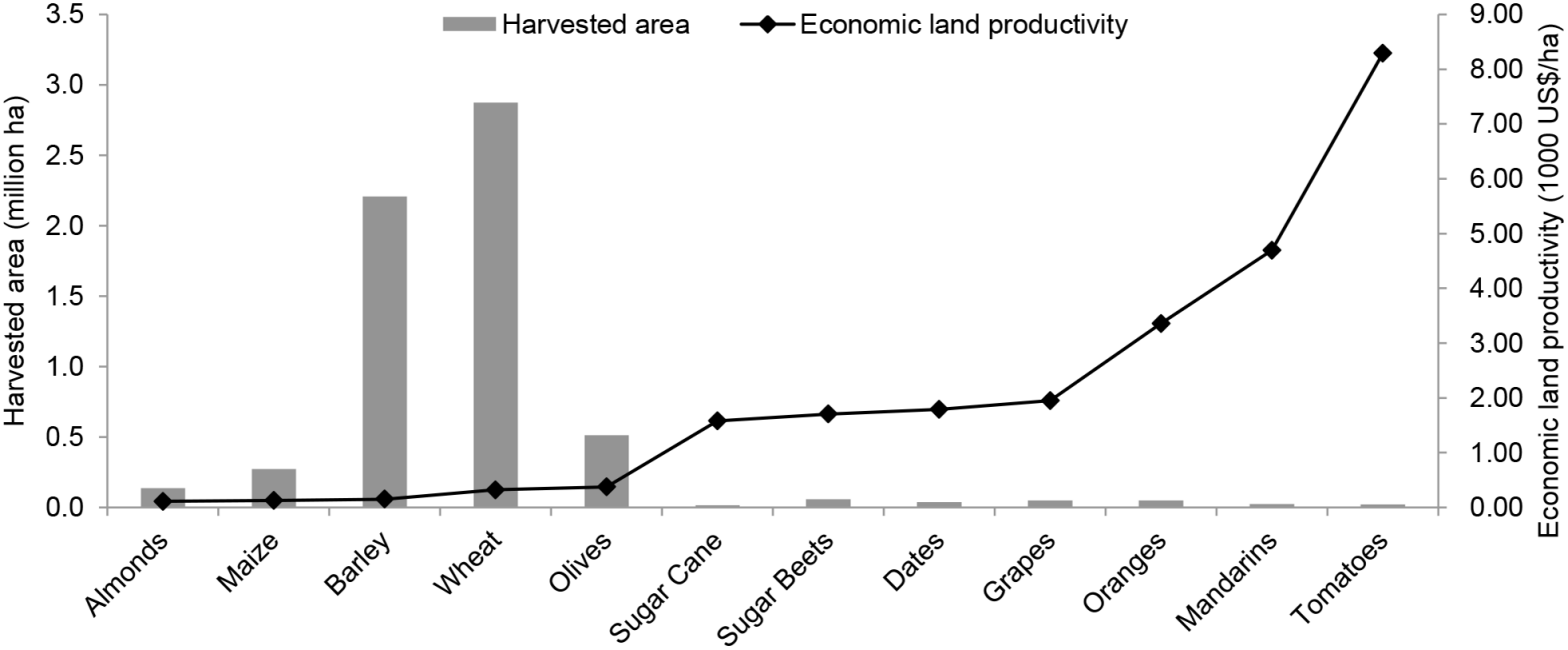
Economic land productivity and harvested area of main crops in Morocco. Period: 1996–2005. Source: Harvested area and yield from Mekonnen and Hoekstra [14], producer prices from FAO [18].

The five crops that consumed the most green plus blue water in the period 1996–2005 are the crops with the lowest economic water productivity, ranging from 0.08 US$/m^3^ for wheat to only 0.02 US$/m^3^ for almonds (Figure 2). Production of tomatoes yielded 22 times more value per drop than production of wheat. The same five crops also have the lowest economic land productivity, ranging from 375 US$/ha for olives to 112 US$/ha for almonds (Figure 4). The highest value per hectare cultivated was obtained by production of tomatoes.

### Virtual Water Trade Balance of Morocco

**Figure 5:**
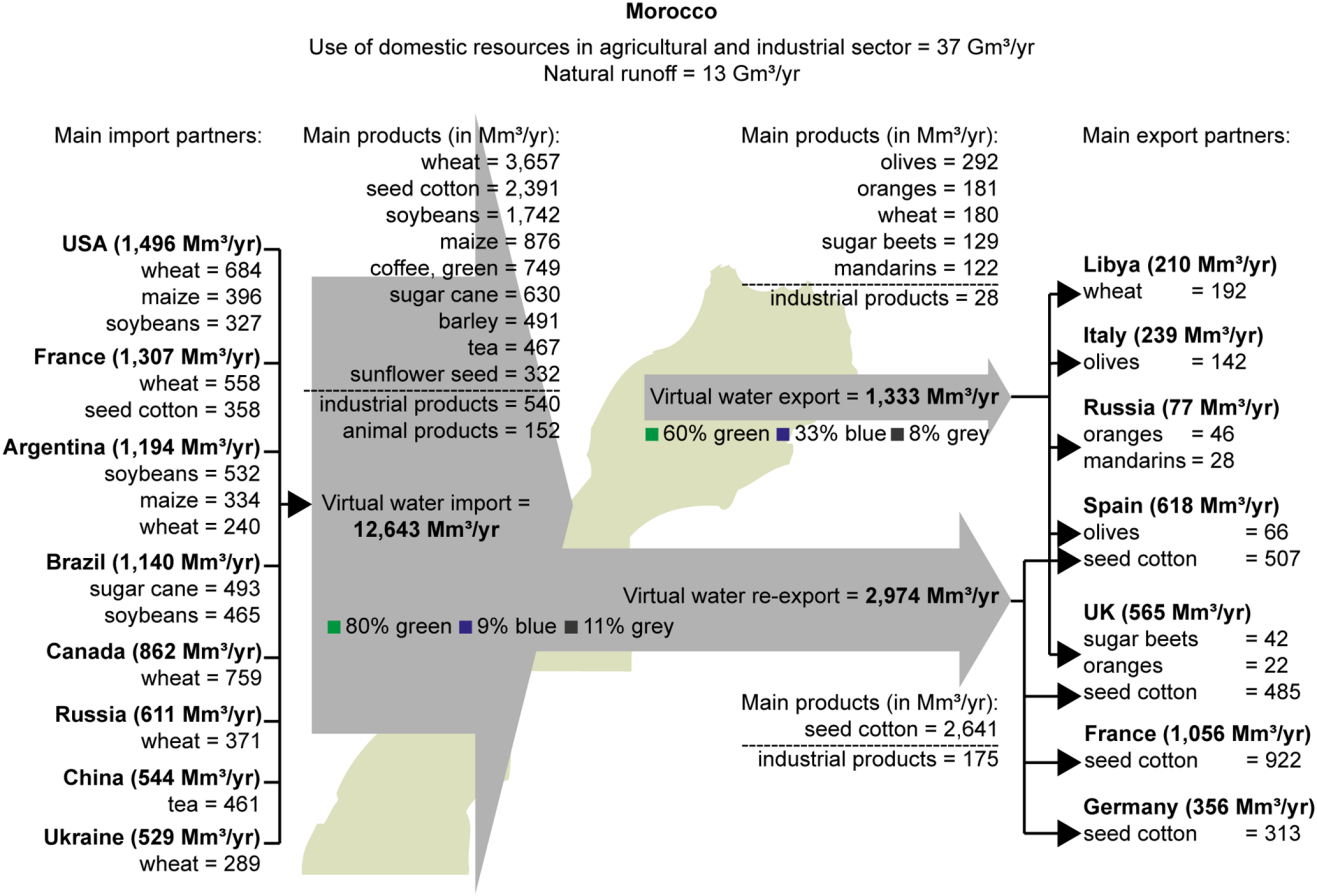
Morocco’s virtual water trade balance related to trade in agricultural and industrial commodities. Period: 1996–2005. Source: Virtual water import and (total) virtual water export from Mekonnen and Hoekstra [13].

Morocco’s virtual water trade balance for the period 1996–2005 is shown in Figure 5. Virtual water import exceeds virtual water export, which makes Morocco a net virtual water importer. Only 31% of the virtual water export originates from Morocco’s water resources, the other 69% is related to re-export of imported virtual water. By import of products instead of producing them domestically, Morocco saved 27.8 Gm^3^/yr (75% green, 21% blue and 4% grey) of domestic water in the period 1996–2005, equivalent to 72% of the water footprint within Morocco.

The value of the total virtual water imported in the period 1996–2005 was 12.4 billion US$/yr. Import of industrial products accounted for 83%, import of crop products for 16% and import of animal products for 1%. The average cost of imported commodities per unit of virtual water imported was 0.98 US$/m^3^. The value of the total virtual water exported in this period was 7.1 billion US$/yr (industrial products: 51%, crop products: 48%, animal products: 1%). The average earning of exported commodities per unit of virtual water exported was 1.66 US$/m^3^.

The total volume of Morocco’s water virtually exported out of the country (i.e. excluding re-export) in the period 1996–2005 is estimated at 1,333 Mm^3^/yr. This means that about 4% of the water used in Morocco’s agricultural and industrial sector is used for making export products. The remainder is used to produce products that are consumed by the inhabitants of Morocco. Virtual export of blue water from Morocco’s resources was 435 Mm^3^/yr, which is to equivalent 3.4% of long-term average natural runoff (13 Gm^3^/yr).

Most of the virtual water export from Morocco’s resources returns relatively little foreign currency per unit of virtual water exported. Export of crop products had the largest share in the virtual water export from Morocco’s water resources (1,305 Mm^3^/yr), returning 0.87 US$/m^3^ on average. Specific crop products associated with large virtual water export from Moroccan origin are olives, oranges, wheat, sugar beets and mandarins. Out of these products, only export of mandarins (122 Mm^3^/yr) returned a value (1.37 US$/m^3^) larger than the average for crop products (0.87 US$/m^3^). On the other hand, virtual water export related to Moroccan tomatoes (24 Mm^3^/yr) yielded 7.13 US$/m^3^.

### Water Footprint versus Water Availability and Waste Assimilation Capacity

**Figure 6:**
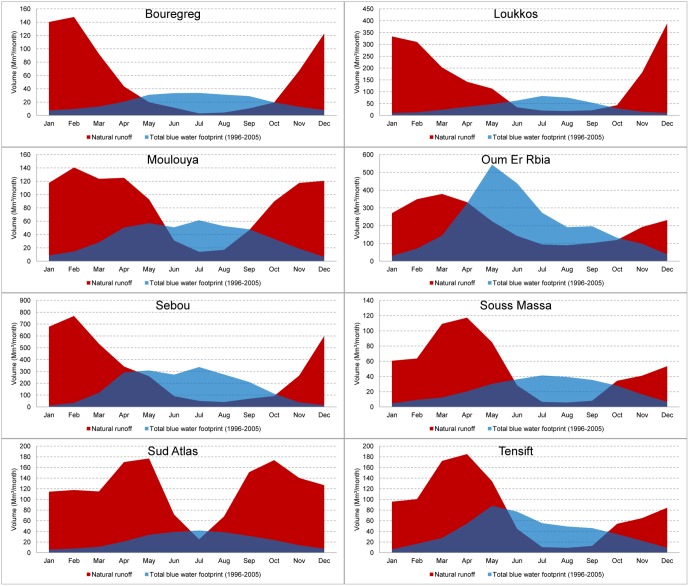
Total blue water footprint and natural runoff per river basin. Period of blue water footprint: 1996–2005. Natural runoff is estimated as the long-term average inflow of reservoirs. It is considered undepleted runoff, since large-scale blue water withdrawals come from the reservoirs. The estimates can be considered conservative, because net precipitation in areas downstream of reservoirs is not included.

Blue water scarcity manifests itself in specific months of the year (Figure 6; Table 4). The average monthly water scarcity indicates severe water scarcity, more severe than annual (total) water scarcity values suggest. In all basins, the total blue water footprint exceeds natural runoff during a significant period of the year. In the months June, July and August, severe water scarcity occurs in all river basins. Crops with a large blue water footprint in July are: sugar beets in Oum Er Rbia and Sebou; grapes in the basins of Sud Atlas, Souss Massa and Oum Er Rbia; dates in Oum Er Rbia and Sebou; sunflower seed in the Sebou basin; maize in the basin of Oum Er Rbia. Demand for potable water peaks in the months June, July and August due to tourism and evaporation from storage reservoirs is large in these months due to the strong evaporative power of the atmosphere. Annual runoff in the Oum Er Rbia basin is almost completely consumed (inter-basin water transfers not yet considered), which raises the question whether it is wise to export water out of this basin to the basins of Bouregreg and Tensift as is common practice.

**Table 4:** Blue water scarcity per river basin.

River basin	Jan	Feb	Mar	Apr	May	Jun	Jul	Aug	Sep	Oct	Nov	Dec	Tot	Avg
Bouregreg	0.05	0.06	0.14	0.47	1.57	2.89	11.3	7.30	2.78	1.01	0.19	0.06	0.37	2.32
Loukkos	0.03	0.04	0.12	0.25	0.42	1.85	4.04	4.11	2.49	0.69	0.08	0.02	0.25	1.18
Moulouya	0.07	0.10	0.23	0.40	0.62	1.65	4.41	3.09	1.03	0.37	0.16	0.05	0.41	1.02
Oum Er Rbia	0.11	0.20	0.38	0.98	2.42	3.08	2.91	2.14	1.93	1.10	0.51	0.16	0.98	1.33
Sebou	0.02	0.04	0.22	0.86	1.19	3.01	6.66	6.72	3.05	1.21	0.14	0.02	0.53	1.93
Souss Massa	0.07	0.14	0.11	0.17	0.36	1.28	6.35	6.82	4.45	0.81	0.40	0.12	0.46	1.76
Sud Atlas	0.05	0.07	0.09	0.12	0.19	0.54	1.67	0.56	0.21	0.14	0.10	0.06	0.19	0.32
Tensift	0.06	0.16	0.16	0.29	0.66	1.72	5.39	5.40	3.66	0.64	0.34	0.11	0.50	1.55
Total	0.05	0.09	0.22	0.56	1.03	2.23	4.15	2.98	1.55	0.66	0.22	0.06	0.52	1.15

Blue water scarcity is defined as the ratio of the total blue water footprint in a catchment over the natural runoff in that catchment. Classification: low blue water scarcity (<0.20); moderate blue water scarcity (0.20–0.30); significant blue water scarcity (0.30–0.40); severe water scarcity (>0.40).

The total groundwater footprint in Morocco constitutes about half of the country’s groundwater availability (Table 5). Groundwater stress is severe in all river basins, except for the basins of Loukkos and Sud Atlas. In the Bouregreg basin, the annual groundwater footprint exceeds annual groundwater availability. As confirmed in the 2012 river basin plan for this basin, most of the aquifers in this basin are indeed overexploited, especially the main aquifers of Berrechid and Chaouia côtière.

**Table 5:** Blue water scarcity related to groundwater.

River basin	Groundwater footprint(Mm^3^/yr)	Groundwater availability (1996–2005)(Mm^3^/yr)	Blue waterscarcity (−)	Level of water scarcity
Bouregreg	106	66	1.60	Severe
Tensift	259	262	0.99	Severe
Oum Er Rbia	510	667	0.77	Severe
Souss Massa	219	349	0.63	Severe
Sebou	689	1,502	0.46	Severe
Moulouya	144	351	0.41	Severe
Loukkos	93	377	0.25	Moderate
Sud Atlas	137	697	0.20	Moderate
Total	2,159	4,347		

Basins are sorted top-down from highest to lowest scarcity.

In the Bouregreg basin there is no waste assimilation capacity of the groundwater left (because the blue groundwater footprint exceeds groundwater availability), which results in an infinite water pollution level (Table 6). In the basins of Tensift and Oum Er Rbia, waste assimilation capacity of the groundwater is also exceeded, even by 43 times the natural groundwater availability in the Tensift basin. These findings correspond with figures reported in the river basin plans for these three basins, which indicate severely high nitrate concentrations in the groundwater (at some measurement stations exceeding the maximum permissible limit in drinking water), mainly caused by diffuse nitrate pollution by the irrational use of nitrogen fertilizers, but in the case of the Sahel-Doukkala aquifer in the Oum Er Rbia basin also by the infiltration of untreated domestic wastewater.

**Table 6:** Water pollution level related to nitrate-nitrogen in groundwater.

River basin	Grey water footprint ofcrop production(1996–2005) (Mm^3^/yr)	Actual groundwateravailability/Wasteassimilation capacity (Mm^3^/yr)	Water pollution level (−)	Waste assimilation capacity exceeded?
Bouregreg	148	0	∞	Yes
Tensift	129	3	43.2	Yes
Oum Er Rbia	435	157	2.78	Yes
Sebou	428	813	0.53	No
Moulouya	99	207	0.48	No
Souss Massa	51	130	0.39	No
Loukkos	63	284	0.22	No
Sud Atlas	25	560	0.04	No
Total	1,378	2,188	0.63	No

Basins are sorted top-down from highest to lowest pollution level.

### Reducing the Water Footprint of Crop Production in Morocco

The regional differences in crop water use (Figure 3) provide an opportunity for reduction of the water footprint of crop production in Morocco. Potential water savings (green plus blue) are in the order of 1.9 and 1.2 billion m^3^ per year when all crops (case A) and when only annual crops (case B) are relocated over the river basins, respectively (Table 7). Blue water savings are 1,276 Mm^3^/yr in case A and 697 Mm^3^/yr in case B. These are significant savings when put in the context of Morocco’s national water strategy, which includes actions plans to mobilize 1.7 billion m^3^/yr by 2030 through the construction of 60 large and 1000 small local dams and an additional 0.8 billion m^3^/yr with the North-South inter-basin water transfer [1].

**Table 7:** Potential water savings by partial relocation of crop production per crop.

		Partial relocationconsidered for all crops*		Partial relocationconsidered for annual crops only**	
	Base casegreen plusblue waterfootprint (Mm^3^/yr)	Saving (green+blue)(Mm^3^/yr)	Relative saving (%)	Saving (green+blue)(Mm^3^/yr)	Relative saving (%)
Almonds	641	14	2%	0	0%
Barley	6,787	–116	–2%	–202	–3%
Dates	449	131	29%	0	0%
Grapes	367	183	50%	0	0%
Maize	1,148	939	82%	939	82%
Olives	2,951	58	2%	0	0%
Oranges	440	15	3%	0	0%
Sugar Beets	353	157	44%	157	44%
Sugar Cane	200	91	46%	0	0%
Mandarins	209	7	3%	0	0%
Tomatoes	99	2	2%	2	2%
Wheat	10,981	413	4%	278	3%
Total	24,625	1,896	8%	1,174	5%

*All analysed crops are: almonds, barley, dates, grapes, maize, olives, oranges, sugar beets, sugar cane, mandarins, tomatoes and wheat.

**Annual crops are: barley, maize, sugar beets, tomatoes and wheat.

Largest potential water savings can be obtained by partial relocation of the production of maize and wheat (Table 7), particularly by moving maize production from the Oum Er Rbia basin to the Moulouya basin and wheat production from the Bouregreg basin to the basin of Sebou. Partial relocation of crop production in case A results in decreased water footprints (green plus blue) in all basins, except for the basin of Bouregreg where the water footprint increases (Table 8). In case B, the water footprints in the basins Bouregreg, Sebou and Loukkos increase, particularly due to increased wheat production in these basins, while the water footprints in the other basins decrease. Precipitation in the basins of Sebou and Loukkos is generally larger than in other parts of Morocco [1].

**Table 8:** Potential water savings by partial relocation of crop production per river basin.

		Partial relocationconsidered for all crops		Partial relocationconsidered for annual crops only**	
	Base case greenplus blue waterfootprint (Mm^3^/yr)	Saving (green+blue)(Mm^3^/yr)	Relative saving (%)	Saving (green+blue)(Mm^3^/yr)	Relative saving (%)
Sud Atlas	306	189	62%	12	4%
Souss Massa	903	175	19%	14	2%
Tensift	2,525	388	15%	124	5%
Oum Er Rbia	8,498	1,229	14%	821	10%
Bouregreg	2,813	−994	–35%	–95	–3%
Moulouya	1,737	605	35%	412	24%
Sebou	6,905	154	2%	–95	–1%
Loukkos	939	151	16%	–19	–2%
Total	24,625	1,896	8%	1,174	5%

*All analysed crops are: almonds, barley, dates, grapes, maize, olives, oranges, sugar beets, sugar cane, mandarins, tomatoes and wheat.

**Annual crops are: barley, maize, sugar beets, tomatoes and wheat.

Reducing the water footprints of crops to benchmark levels leads to a potential green plus blue water saving of 2,768 Mm^3^/yr, a reduction of 11% (Table 9). Fifty-two per cent of this saving is related to reduced water footprints (i.e. improved water productivities) in the Sebou basin alone. Largest potential water savings are associated with reducing the water footprints of cereals, especially wheat. Blue water savings are estimated at 422 Mm^3^/yr and are largest in the basins of Sebou and Oum Er Rbia.

**Table 9:** Potential water savings by benchmarking water productivities of main crops* (in Mm^3^/yr).

	Sud Atlas	Souss Massa	Tensift	Oum Er Rbia	Bouregreg	Moulouya	Sebou	Loukkos	Total
Almonds	0	2	1	0	3	0	8	0	14
Barley	0	0	0	100	158	222	238	0	717
Dates	0	0	0	10	0	4	48	0	63
Grapes	0	20	0	5	0	0	18	4	48
Maize	0	13	0	175	32	0	33	0	254
Olives	0	9	4	0	10	0	35	0	59
Oranges	0	1	1	0	1	0	6	0	9
Sugar Beets	0	0	0	0	0	0	70	4	73
Sugar Cane	0	0	0	0	0	0	79	10	89
Mandarins	0	1	0	0	0	0	3	0	4
Tomatoes	0	0	0	0	1	0	1	0	3
Wheat	0	14	0	102	417	0	904	0	1,436
Total (gn+bl)	0	60	6	392	623	226	1,444	18	2,768
Total (blue)**	0	23	2	113	11	2	258	12	422
Total (blue) (% of natural runoff)	0%	4%	0%	4%	2%	0%	7%	1%	3%

*Analysed crops are: almonds, barley, dates, grapes, maize, olives, oranges, sugar beets, sugar cane, mandarins, tomatoes and wheat.

**Assuming that the green/blue water ratio remains the same for all basins and crops.

### Added Value of Water Footprint Assessment for Morocco’s Water Policy

Several insights and response options emerged from the Water Footprint Assessment, which are currently not considered in the national water strategy of Morocco and the country’s river basin plans. They include:

- New insights in the water balance of Morocco and the country’s main river basins:
  - The evaporative losses from storage reservoirs account for a significant part of the blue water footprint within Morocco. This sheds fresh light on the national water strategy that proposes to build another 60 large and 1000 small dams by 2030.
  - Blue water scarcity on a monthly scale is severe and hidden by annual analysis of demand versus supply, which is the common scale of analysis in Morocco’s river basin plans.
- New insights in how economically efficient water and land resources are used:
  - Analysis of the economic value of crop products per unit of water and land used in the period 1996–2005 indicate that agricultural policy may be better brought in line with water policy by reconsidering which crops to grow.
  - It is shown that the export policy in this period was not optimal from a water-economics point of view, which raises the question whether the foreign income generated by export covers the direct and indirect costs of mobilization and (over) exploitation of Morocco’s water resources. This might not be the case considering the costs of the construction and maintenance of the large dams and intra- and inter-basin water transfers in the country and the costs associated with the negative externalities of water (over) consumption, such as the salt-intrusion in Morocco’s coastal aquifers.
- New response options to reduce the water footprint of crop production:
  - Analysis of the water footprint of the main crops in Morocco and its variation across the river basins offers new ways of looking at reducing water consumption in the agricultural sector. The estimated potential water savings by partial relocation of crops to basins where they consume less water and by reducing water footprints of crops down to benchmark levels are significant compared to demand reducing and supply increasing measures considered in the national water strategy of Morocco.

## Discussion

Morocco’s water footprint is mostly green (77%). This underlines the importance of green water resources, also (or especially) in semi-arid countries with a high dependency on blue water, and is in line with other studies showing the dominance of the green over the blue water flow in Africa (and most of the world) [25], [26]. The relevance of the green water footprint should not be underestimated. Although rain is free and evaporation happens anyway, green water that is used for one purpose cannot be used for another purpose [27].

Storage reservoir evaporation accounts for a significant share (13%) in the blue water footprint in Morocco. The need for seasonal storage of water is evident given the large mismatch in natural runoff and water demand (Figure 6). However, the large evaporation from reservoirs shows that these should be seen as water consumers, besides their role in water supply. This water footprint can ultimately be linked to the end-purpose of the reservoir, which for most cases in Morocco is primarily serving irrigated agriculture. Therefore, to reduce the need for seasonal storage and hence the water footprint of storage reservoirs, it would be worthwhile to take the timing of crop water demands with respect to natural water availability into account in deciding which crops or crop varieties to grow. Furthermore, local alternatives to the large surface water reservoirs are groundwater dams, which enhance underground water storage in alluvial aquifers and thereby loose less water by evaporation [28].

Our analysis shows that from a strictly water-economics point of view it would be worthwhile to reconsider which crops to grow in Morocco (due to the low value in US$/m^3^ and US$/ha for some crops compared to others). In practice, the choice of which crops to produce is part of the national strategy regarding food security and of course closely linked to the demand for crops (national and global). Nevertheless, we consider it useful and important to analyse economic water and land productivities (as done in this study) in addition to these considerations. Especially for water-short countries as Morocco it is relevant to evaluate the economic efficiency of water allocation. This also relates to the question whether the foreign income generated by export products, which have a footprint on national resources, outweighs the direct and indirect costs associated with the resource use.

### Uncertainties and Limitations

The water footprint of crop production is largely influenced by the input data used and assumptions made by Mekonnen and Hoekstra [14] and can easily contain an uncertainty of ±20% [14], [29], [30]. The calculated economic water and land productivities of crops are, apart from the water footprints and yields, dependent on the producer prices. Variations in these prices largely influence the economic water and land productivity of crops. The water footprints of industrial production and domestic water supply are very sensitive to the consumptive fractions applied.

Although figures on water availability are based on data from the river basin plans and the Ministry EMWE (unpublished data 2013), the way they are estimated exactly is often unclear and so is the uncertainty in them. Since natural runoff is estimated as the inflow of reservoirs (thus excluding small-scale local abstractions upstream) and net precipitation in areas downstream of reservoirs is not included, the estimates of natural runoff can be considered conservative.

In general, the river basin plans indicate larger pressure on groundwater resources than suggested in this study. This might be caused by the fact that the river basin plans include more recent withdrawals and because the unit of analysis in this study (river basin agency action zone) is larger than the unit used in the river basin plans (individual aquifers), whereby in this study overexploitation of one aquifer might be masked by low exploitation of another. Also local groundwater pollution according to the river basin plans is sometimes worse than the water pollution level estimated here. This could be explained by the fact that the water quality measurements recorded in the basin plans are partly more recent and are measured at specific points, whereas this study considered homogeneous distribution of nitrates in the groundwater.

Given the uncertainties and limitations of the study, the presented water footprint estimates and water scarcity values should be interpreted with care. Nevertheless, the order of magnitude of the estimates in this study gives a good indication to which activities and crops Morocco’s water resources are allocated, in which months and basins the water footprints are relatively large or small and where and when this leads to highest water scarcity.

Uncertainties in the estimated potential savings by relocation of crop production and reducing the water footprints of crops to benchmark levels are closely linked to the uncertainties in the estimates of the water footprint of crop production and the results should be interpreted carefully. However, the order of magnitude of the estimated savings gives a rough indication of the potential of these measures. When considering relocation of crop production it is necessary to assess how the green and blue water footprints of crops manifest themselves on a monthly scale. This study looked at annual water savings, but the associated relocation of crops might well aggravate monthly water scarcity in some river basins. Furthermore, the feasibility and desirability of relocation of crop production are of course largely determined by social and economic factors which are not considered in this study.

## Conclusion

The study finds that: (i) evaporation from storage reservoirs is the second largest form of blue water consumption in Morocco, after irrigated crop production; (ii) Morocco’s water and land resources are mainly used to produce relatively low-value (in US$/m^3^ and US$/ha) crops such as cereals, olives and almonds; (iii) most of the virtual water export from Morocco relates to the export of products with a relatively low economic water productivity (in US$/m^3^); (iv) blue water scarcity on a monthly scale is severe in all river basins and pressure on groundwater resources by abstractions and nitrate pollution is considerable in most basins; (v) the estimated potential water savings by partial relocation of crops to basins where they consume less water and by reducing water footprints of crops down to benchmark levels are significant compared to demand reducing and supply increasing measures considered in Morocco’s national water strategy.

On the basis of these new insights and response options it is concluded that Water Footprint Assessment has an added value for national water policy in Morocco. Water Footprint Assessment forces to look at end-users and -purposes of freshwater, which is key in determining efficient and equitable water allocation within the boundaries of what is environmentally sustainable, both on the river basin and on the national level. This is especially relevant for water-scarce countries such as Morocco. Furthermore, considering the green and grey components of a water footprint provides new perspectives on blue water scarcity, because pressure on blue water resources might be reduced by more efficient use of green water and by less pollution.
